# Inosine induces acute hyperuricaemia in rhesus monkey (*Macaca mulatta*) as a potential disease animal model

**DOI:** 10.1080/13880209.2020.1871373

**Published:** 2021-03-14

**Authors:** Dong-hong Tang, Chen-yun Wang, Xi Huang, Hong-kun Yi, Zhe-li Li, Kai-li Ma, You-song Ye, Jian-wen Zhang

**Affiliations:** a Medical Primate Research Center of China, Institute of Medical Biology, Chinese Academy of Medical Sciences/Peking Union Medical College, Kunming, China; b KPC Pharmaceuticals Inc., Kunming, China

**Keywords:** Inosine, uric acid, hyperuricaemia, animal model

## Abstract

**Context:**

The uric acid metabolism pathway is more similar in primates and humans than in rodents. However, there are no reports of using primates to establish animal models of hyperuricaemia (HUA).

**Objectives:**

To establish an animal model highly related to HUA in humans.

**Materials and methods:**

Inosine (75, 100 and 200 mg/kg) was intraperitoneally administered to adult male rhesus monkeys (*n* = 5/group). Blood samples were collected over 8 h, and serum uric acid (SUA) level was determined using commercial assay kits. XO and PNP expression in the liver and URAT1, OAT4 and ABCG2 expression in the kidneys were examined by qPCR and Western blotting to assess the effects of inosine on purine and uric acid metabolism. The validity of the acute HUA model was assessed using ulodesine, allopurinol and febuxostat.

**Results:**

Inosine (200 mg/kg) effectively increased the SUA level in rhesus monkeys from 51.77 ± 14.48 at 0 h to 178.32 ± 14.47 μmol/L within 30 min and to peak levels (201.41 ± 42.73 μmol/L) at 1 h. PNP mRNA level was increased, whereas XO mRNA and protein levels in the liver were decreased by the inosine group compared with those in the control group. No changes in mRNA and protein levels of the renal uric acid transporter were observed. Ulodesine, allopurinol and febuxostat eliminated the inosine-induced elevation in SUA in tested monkeys.

**Conclusions:**

An acute HUA animal model with high reproducibility was induced; it can be applied to evaluate new anti-HUA drugs *in vivo* and explore the disease pathogenesis.

## Introduction

Hyperuricaemia (HUA) occurs because of purine metabolism aberrations and is typically characterized by increased uric acid formation or reduced uric acid excretion. HUA is defined as a serum urate concentration that allows urate saturation in bodily fluids (>7.0 mg/dL) (Roddy and Choi 2014). The incidence of HUA, which has currently increased (Roddy and Choi 2014), is approximately 13.3% (19.4% in men and 7.9% in women) in the Chinese population (Liu et al. 2015). Although the condition is more common in middle-aged or older men and postmenopausal women, the age at disease onset has been decreasing in recent years (Liu et al. 2015). Uric acid increases the risk of gout and cardiovascular disorders, nephrolithiasis, diabetes, obesity, dyslipidaemia (Feig 2014; Abeles 2015), chronic kidney diseases and metabolic syndromes (Feig 2014; Abeles 2015; Hahn et al. 2017). Therefore, a reliable animal model is urgently needed to screen drugs against HUA and investigate its pathophysiology (Zhu et al. 2017).

Uric acid production and metabolism are complex processes involving various factors that regulate uric acid production in the liver and reabsorption or excretion from the kidneys and gut (Mori and Percudani 2016; Yun et al. 2017). The balance of these processes maintains uric acid homeostasis. Several enzymes are involved in the conversion of the purine nucleotides adenine and guanine to uric acid, including purine nucleoside phosphorylase (PNP) and xanthine oxidase (XO), which catalyse the formation of xanthine, and subsequently, uric acid, respectively (Magness et al. 2005; Yan et al. 2011). Furthermore, these enzymes catalyse the conversion of adenosine and inosine, which are precursors of purine metabolism. Similarly, the conversion of guanosine is catalysed by these enzymes to guanine, xanthine and, finally, to uric acid. Therefore, an abnormal activity of these enzymes can lead to increased uric acid production, leading to HUA (Ishikawa et al. 2013).

Uric acid is excreted and reabsorbed from the kidneys and gut via uric acid transporters, whose aberrant expression is linked to HUA. Urate transporter 1 (URAT1, encoded by *SLC22A12*) is an important organic anion transporter that maintains uric acid homeostasis. It is located in the brush border of proximal tubular epithelial cells and mainly mediates the reabsorption of uric acid. Intracellular urate is released through glucose transporter 9 (GLUT9, encoded by *SLC2A9*). A multiple specific anion transporter, organic anion transporter 4 (encoded by *SLC22A11*), is located in the apical membrane of epithelial cells and has been demonstrated to promote urate reabsorption (Wright et al. 2010; Xu et al. 2017). ABCG2, a half-transporter protein with an ATP-binding cassette, has also been genetically linked to serum uric acid (SUA) level, HUA and gout (Wright et al. 2010; Xu et al. 2017). ABCG2 mediates renal and/or extra-renal urate excretion as a high-capacity exporter and is abundantly expressed in the proximal tubule cells, at the apical membrane, and in hepatocytes (Woodward et al. 2009; Hosomi et al. 2012).

In most mammals, uric acid is degraded by hepatic uricase, i.e., urate oxidase, to allantoin, which is more soluble and more readily excreted in the urine. However, in humans and primates, the enzyme was reportedly lost during evolution (Fujiwara et al. 1987; Oda et al. 2002) for unknown reasons. Therefore, the uric acid metabolic pathway in primates is more similar to that of humans, but differs from that of rodents. Accordingly, rodents generally have limited applications as HUA animal models, but are nonetheless commonly used (Zhu et al. 2017). Purine metabolism ends at uric acid formation in chickens and in other birds, and thus, these birds have also been used as HUA animal models; however, there exists a large taxonomic distance between these birds and humans. For these reasons, a primate model of human HUA would be useful. Indeed, primates are considered as ideal, and sometimes the only suitable animal model, from which results can be directly correlated with human results because of the high similarities in the metabolic, immune and nervous systems between humans and primates. However, primates are costly and complex to manage (Zhu et al. 2017) and are rarely used, particularly as models of HUA.

In this study, we induced HUA in the rhesus monkey, an Old-World non-human primate, via intraperitoneal injection of inosine, a key precursor in purine metabolism. The effects of three highly specific inhibitors of uric acid synthesis, ulodesine, allopurinol and febuxostat, were evaluated in this model, along with the levels of serum urate; hepatic *PNP* and *XO*; and renal URAT1 (*SLC22A11*), GLUT9 (*SLC2A9*), *ABCG2* and OAT4 (*SLC22A13*).

## Materials and methods

### Drugs and reagents

Inosine was purchased from Sigma-Aldrich (lot no. SLBS1811V, PCode 1002403125, St. Louis, MO). Allopurinol was purchased from Nanjing Dolai Biological (cas: 315-30-0, Nanjing, China). Ulodesine (lot no. 20150727) and febuxostat (lot no. 2014-FB-0902) were procured from WuXi AppTec, WuXi, Shanghai, China). The uric acid assay kit was purchased from Nanjing Jiancheng Biological Engineering Institute (lot no: 20170701, Nanjing, China), and the transcriptor first-strand cDNA synthesis kit (catalogue no. 04897030001) was obtained from Roche (Basel, Switzerland). Premix Taq™ (code no. RR003A) was purchased from Takara Biotechnology (Dalian, China), and TriPure isolation reagent (lot # 94013920) was purchased from Roche (Basel, Switzerland). Ketamine hydrochloride (lot no. 161201) was purchased from Jiangsu Zhongmu Beikang Pharmaceutical (Taizhou, China). Antibodies against XO (ab109235-10000), PNP (ab109559), URAT1 (RST, Ab181237), GLUT9 (Ab223470) and ABCG2 (Ab207732) were purchased from Abcam (Cambridge, UK). The antibody against OAT4 (SLC22A11, A7816) was purchased from ABclonal Biotechnology (Wuhan, China). The RIPA lysis buffer and BCA protein concentration assay kit were purchased from Beyotime (P0013B, P009-1, Shanghai, China). The polyvinylidene fluoride membrane was purchased from Millipore (K2MA8350E, Billerica, MA), and all other chemicals were from Sigma-Aldrich (St. Louis, MO).

### Animals and ethics statement

Animal welfare and experimental procedures were conducted in accordance with the Guide for the Care and Use of Laboratory Animals (National Research Council Institute for Laboratory Animal, R. (1996). Washington (DC), National Academies Press (US). And all efforts were made to minimize animal suffering. In this study, to determine the changes in liver XO, PNP, and renal transporter mRNA and protein expression following inosine administration, liver and kidney tissue samples had to obtain. To minizine pain and distress, type-B ultrasound was used for obtaining the fresh tissue samples. After puncture, the needle, which was accurately positioned and not near any major blood vessels such as the portal vein or inferior vena cava, was rapidly withdrawn; hence, the animals recovered quickly with little to no pain and no postoperative bleeding or infection. During the study, no death had occurred, and all animals were fully recovered by the end of this experiment.

### Dose-dependent effects of inosine

Monkeys were used as experimental animals to approach the optimal dose of inosine to induce highly repeatable acute HUA statuses in the test animals. The monkeys were randomly assigned to four groups of five animals each. The control group was intraperitoneally injected with 0.9% saline, whereas the other groups were intraperitoneally injected with inosine in 0.9% saline at doses of 75, 100 and 200 mg/kg. Blood samples were collected at 0, 0.5, 1, 2, 4 and 8 h after administration. Serum was separated by centrifuging the blood samples at 5000×*g* for 15 min at 10 °C; serum SUA level was determined using commercial assay kit.

### Ulodesine and febuxostat activities in monkeys with acute HUA

Monkeys were randomly assigned to four groups of five animals each. The control animals were intraperitoneally injected with 0.9% saline. Experimental groups were intraperitoneally injected with the following: 200 mg/kg inosine in 0.9% saline, 200 mg/kg inosine followed by 2 mg/kg ulodesine and 200 mg/kg inosine followed by 2 mg/kg febuxostat. Blood samples were collected 0, 0.5, 1, 2, 4 and 8 h after administration. Serum was separated by centrifuging the blood samples at 5000×*g* for 15 min at 10 °C, and then stored at −80 °C until use. Serum SUA concentration was determined using commercial kit, according to the manufacturer’s instruction.

### Allopurinol activity in monkeys with acute HUA

Monkeys were randomly assigned to three groups of five animals each. The control animals were injected intraperitoneally with 0.9% saline. The experimental groups were intraperitoneally injected with the following: 200 mg/kg inosine in 0.9% saline and 200 mg/kg inosine followed by 2.5 mg/kg allopurinol. Blood samples were collected 0, 0.5, 1, 4 and 8 h after administration; the SUA level was determined as described above.

### Collection of the liver and kidney biopsies under B-mode ultrasound guidance

Monkeys were randomly assigned to three groups of five animals each and exposed to inosine and allopurinol as described in the previous section. Approximately, 20 mg of the liver and kidney tissues (based on volume estimates) was obtained under B-mode ultrasound guidance at 1 h after inosine injection. Briefly, the animals were anaesthetized by intramuscular injection of 5–10 mg/kg ketamine hydrochloride. Their abdomens were then shaved and disinfected with iodophor. The liver and kidneys were located by scanning the intercostal area of the right upper quadrant or lateral waist subcostal abdomen using Biosound Esaote MyLab 30CV (Providian, Chagrin Falls, OH) fitted with a 3.5-Hz transducer (Esaote) and fixed to a guide frame. Biopsies were obtained using a biopsy gun with the range set to 15 mm and positioned perpendicularly to the puncture target and away from large vessels such as the portal vein and inferior vena cava. After puncture, the needle was withdrawn quickly. The tissues were stored in liquid nitrogen until the extraction of total RNA and proteins.

### RNA isolation and reverse transcription qRT-PCR

The total RNA was extracted from the liver and kidney biopsies (20 mg) using 1 mL of TriPure (Roche, Basel, Switzerland). RNA integrity was evaluated by 1% agarose electrophoresis, and the samples were stored at −80 °C. Subsequently, 2 μg of RNA was reverse-transcribed using Transcriptor for Strand cDNA Synthesis (Roche, Basel, Switzerland), according to the manufacturer’s protocol. The obtained cDNA was stored at −20 °C. *XO* (GenBank: NM_000379.3), *PNP* (GenBank: NM_001193551), *URAT1* (GenBank: AB738914.1), *GLUT9* (GenBank: NM_020041.2), *OAT4* (GenBank: NM_018484) and *ABCG2* (GenBank: NM_001032919.1) were amplified by quantitative real-time PCR (qRT-PCR) on a *CFX384* Touch™ Real-Time PCR Detection System (Bio-Rad, Hercules, CA). The polymerase chain reaction (PCR) conditions were as follows: initial denaturation for 5 min at 95 °C, and 40 cycles of 5 s at 95 °C and 30 s at 60 °C. Gene expression was quantified by the ΔΔC_t_ method with CXF386 software (Bio-Rad, Hercules, CA), and normalized to *GAPDH* (GenBank: NM_001195426.1) as an internal control. Primers were designed using Primer 5.0 and synthesized by Takara Biotechnology (Kusatsu, Japan) (Table 1).

**Table 1.** qRT-PCR primers and parameters.

Target	Sequence (5′–3′)	Product size (bp)	Annealing temperature (°C)	Cycles
XDH/XO	TCATTTCCGCTGATGATGTTCC	106	60	40
	GCACCAATGATATGCCCAACAC			
PNP	GAGACCATGGAGAACGGATACAC	120	60	40
	CAGACCTCCTAATCCAGAACCAC			
URAT1	TGAGCCATGAGGGAGGAAC	119	60	40
	CCAAAGGCGAACCAGCA			
OAT4	GGTGGCTGATTATTAAGGGCAAAC	122	60	40
	CTTCACGCTGGACATCAGCA			
GLUT9	CCACGCTACCTGCTCTTGGA	75	60	40
	TGCTTTCCCAAGAACGTTGGA			
ABCG2	TTAGCTGCAAGGAAAGATCCAAG	182	60	40
	GTCATAGTTGTTGGAAGGCCGAAAG			

### Western blotting

Protein samples were extracted from the renal cortex tissues and quantified using a BCA protein concentration assay kit. Protein samples were separated on a 10% SDS-PAGE separation gel and transferred onto a 0.45 μm polyvinylidene fluoride membrane. The membranes were blocked with 5% skim milk in TBST buffer (Tris–HCl, NaCl, Tween 20) for 30 min and subsequently incubated with primary and secondary antibodies. Primary antibodies were as follows: rabbit monoclonal antibodies against XO (1:5000; ab109235), rabbit polyclonal antibodies against PNP (1:25,000; ab109559), URAT1 (1:1000; ab7816), GLUT9 (1:1000; ab223470) and OAT4 (1:1000; A7816); and mouse monoclonal antibodies against ABCG2 (1:1000; ab207732) and β-actin (1:5000; ab6276). Reactivity was detected using an anti-rabbit IgG (whole molecule)-peroxidase antibody (1:5000; Sigma A6154, St. Louis, MO) or anti-mouse IgG (whole molecule)-peroxidase antibody (1:5000; Sigma A4416, St. Louis, MO). Immunoreactive bands were detected with ECL reagents, and the densitometry analysis of the immunoblot results was conducted using ImageJ software (National Institutes of Health, Bethesda, MD).

### Statistical analyses

Data are reported as mean ± standard error of mean (SEM), and the groups were compared using GraphPad Prism 8 software (GraphPad, Inc., San Diego, CA) using the one-way analysis of variance followed by Tukey’s post hoc test for multiple comparisons. Differences were considered significant at *p* < 0.05.

## Results

### Dose-dependent effects of inosine on SUA

**Figure 1.**
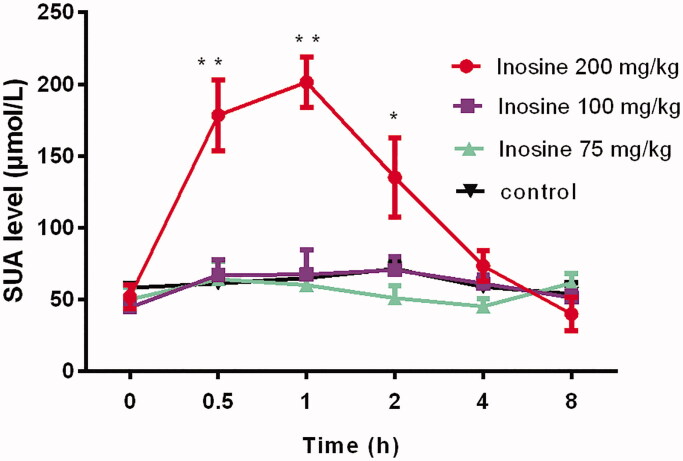
Dose-dependent effects of inosine in rhesus monkeys. Data are presented as mean ± SEM, *n* = 5/group. *p< 0.05; **p< 0.01 vs. control monkeys.

The SUA level increased rapidly within 30 min after injecting a high dose (200 mg/kg) of inosine, which peaked at 1 h, and then decreased gradually, returning to the baseline level within 4 h of inosine administration. The SUA level increased from 51.77 ± 14.48 μmol/L at 0 h to 178.32 ± 14.47, 201.41 ± 42.73 and 134.89 ± 30.1 μmol/L at 0.5, 1 and 2 h, respectively, after inosine administration, compared with that in the control monkeys (*p*< 0.01). However, in the two lower inosine dose groups (75 and 100 mg/kg), stable elevation in the SUA level was not observed at the corresponding time points. Indeed, the injection of 100 and 75 mg/kg inosine did not significantly increase the SUA level at 0.5, 1, 2, 4 and 8 h after administration compared with the injection of saline (Figure 1).

### Ulodesine, febuxostat and allopurinol activities in monkeys with acute HUA

**Figure 2.**
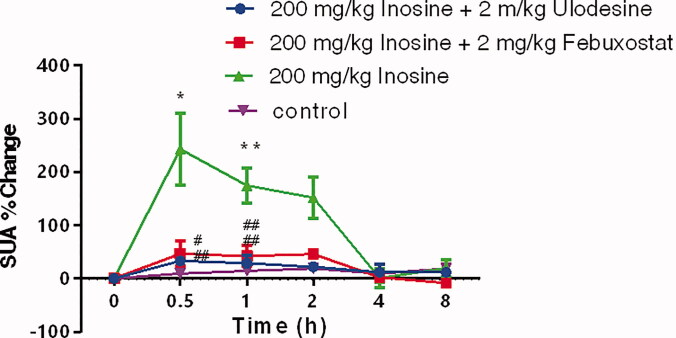
Activity of ulodesine and febuxostat in rhesus monkeys with acute HUA, as measured by the percentage change in SUA 0, 0.5, 1, 2, 4 and 8 h after administration. Data are presented as mean ± SEM, *n* = 5/group. *p< 0.05, **p< 0.01 vs. control monkeys. ^#^*p*< 0.05, ^##^*p*< 0.01 vs. 200 mg/kg inosine-treated HUA monkeys.

**Figure 3.**
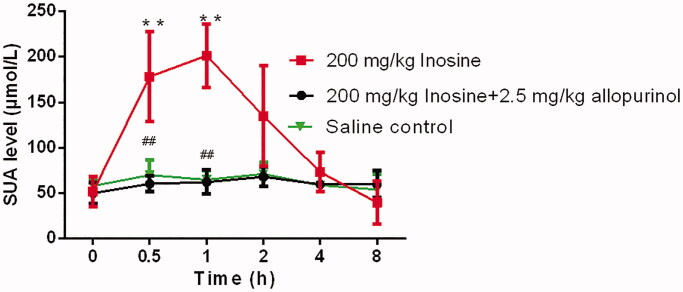
Activity of allopurinol in rhesus monkeys with acute HUA. Data are presented as mean ± SEM, *n* = 5/group. **p< 0.01 vs. control monkeys. ^##^*p*< 0.01 vs. 200 mg/kg inosine-treated HUA monkeys.

The three highly specific inhibitors of uric acid synthesis, ulodesine, allopurinol and febuxostat, reversed the inosine-induced elevation in the SUA level. As shown in Figure 2, the effects of inosine on the SUA level in monkeys, as shown in the plots of the percentage change in SUA levels at 0.5, 1, 2, 4 and 8 h after the induction of HUA, were effectively blocked by ulodesine or febuxostat administration, at fixed doses of 2.0 and 2.0 mg/kg, respectively. Similarly, the administration of 2.5 mg/kg allopurinol effectively reduced the SUA level in monkeys treated with 200 mg/kg inosine (Figure 3).

### mRNA and protein levels of PNP and XO analysed by qRT-PCR and Western blotting in the hepatic tissues

**Figure 4.**
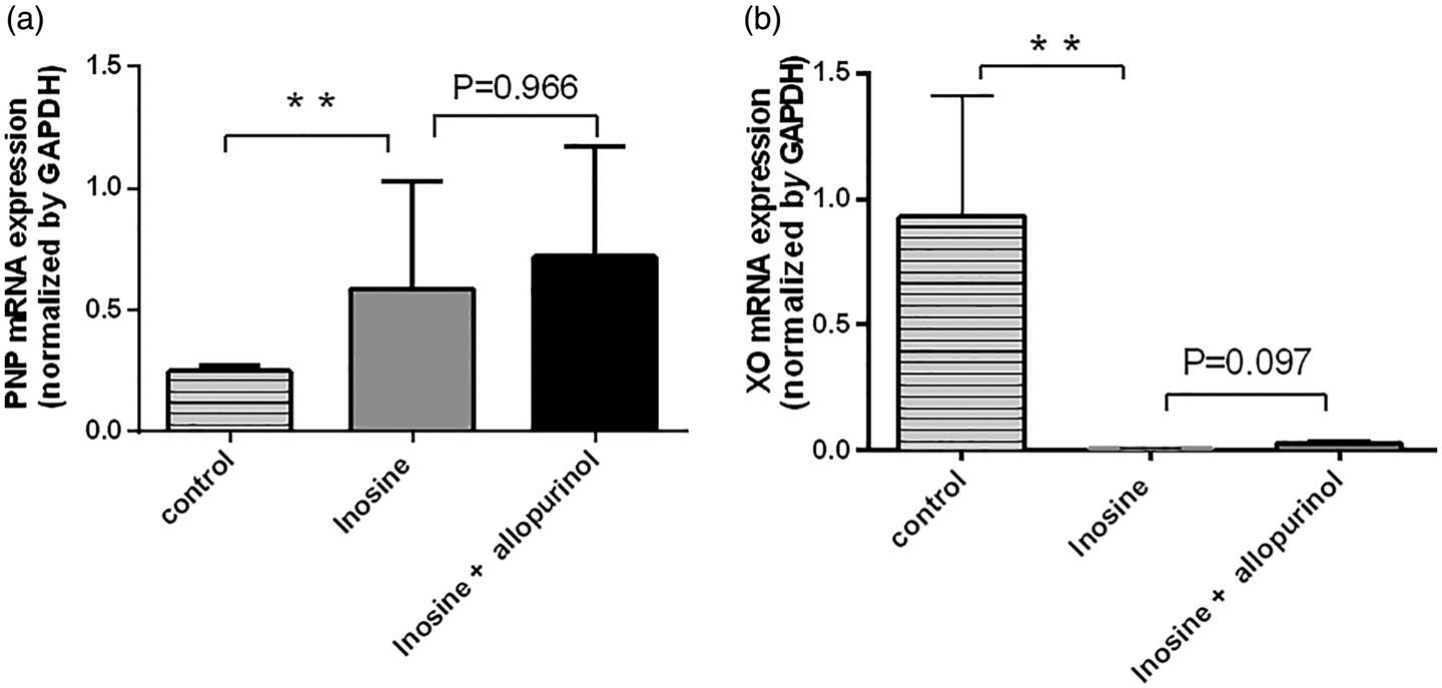
Reverse transcription-qPCR analysis of *PNP* and *XO* in the liver tissues. Data are presented as mean ± SEM, *n* = 5/group. **p< 0.01 vs. control monkeys.

**Figure 5.**
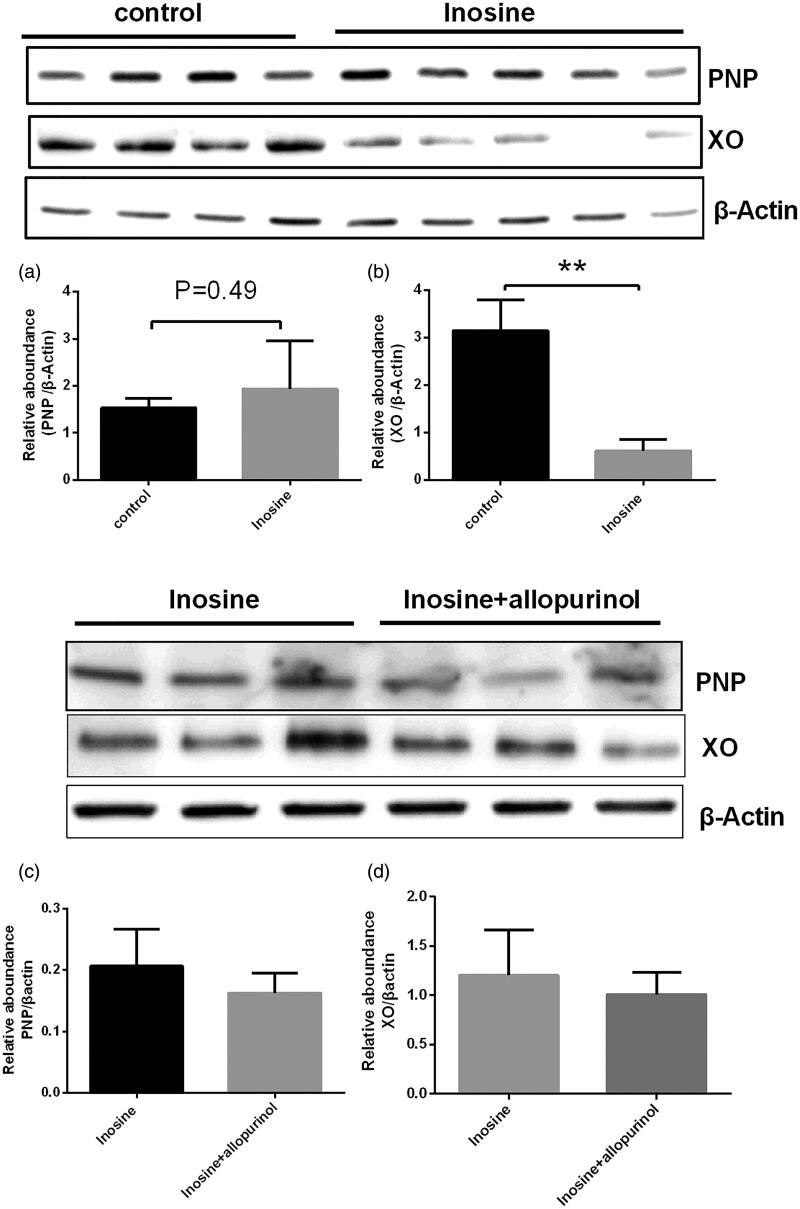
Western blotting of PNP and XO in the liver tissues. Data are presented as mean ± SEM, *n* = 5/group. **p< 0.01 vs. control monkeys.

At 1 h post administration, inosine significantly upregulated *PNP* mRNA expression in the liver (2.36-fold; *p*< 0.05; Figure 4(a)) and significantly downregulated *XO* mRNA expression (93-fold) to a nearly undetectable level (Figure 4(b)) in the liver of monkeys in the treatment group compared with that in the control group. Furthermore, inosine also significantly decreased the protein level of XO in the liver (*p*< 0.05) of animals in the treatment group compared with that in the control group (Figure 5(b)). The protein level of PNP was not significantly increased in the treatment group compared with that in the control group (*p*> 0.05, Figure 5(a)). The Western blotting analysis showed that the protein expression levels of PNP and XO were not significantly decreased after administration of 2.5 mg/kg allopurinol compared with those in the inosine treatment group (*p*> 0.05 Figure 5(c,d)).

### qRT-PCR and Western blotting analyses of the renal transporters URAT1, GLUT9, OAT4 and ABCG2 in renal tissues

**Figure 6.**
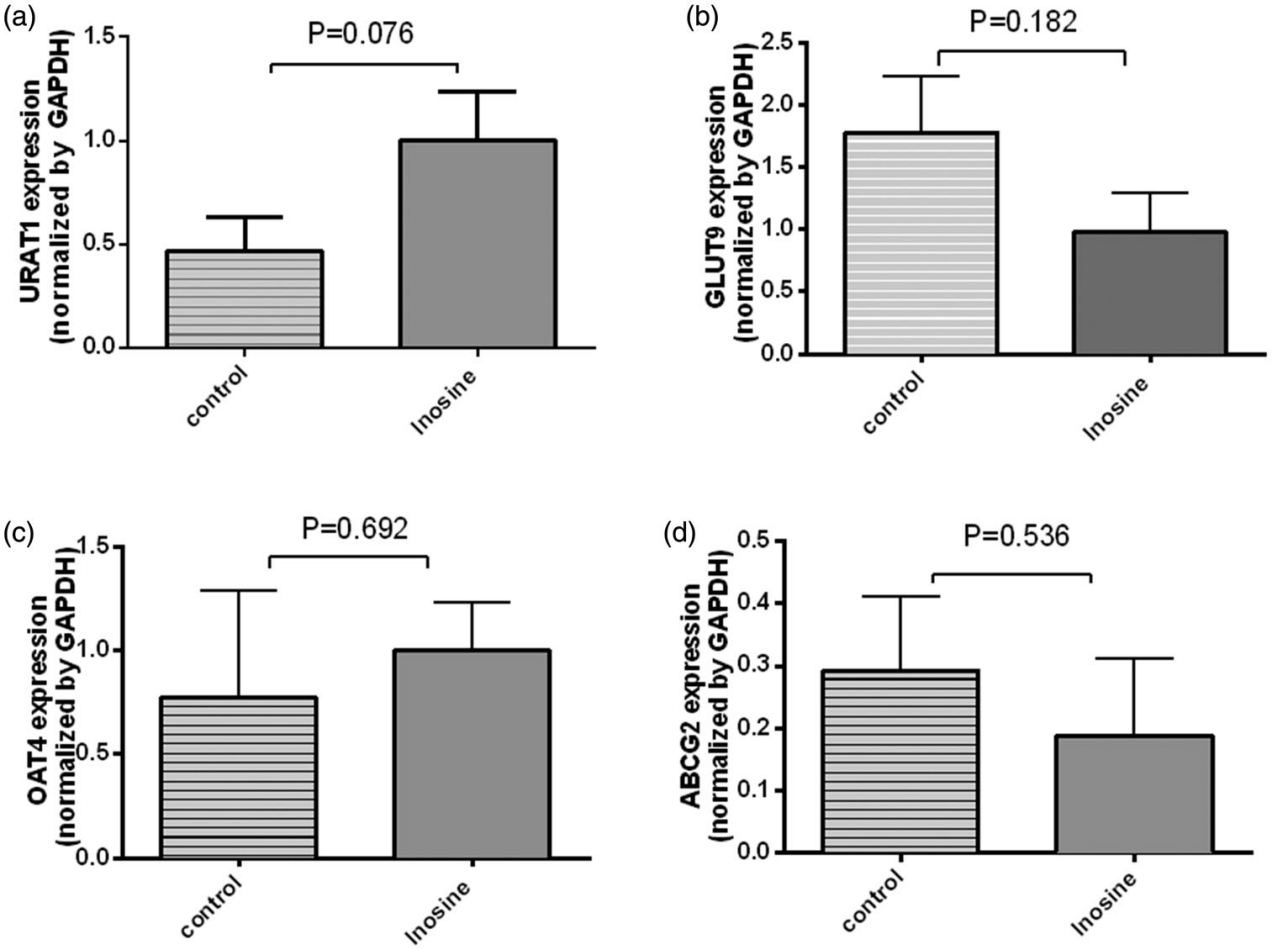
Reverse transcription-qPCR analysis of *URAT1*, *GLUT9*, *OAT4* and *ABCG2* in the kidney cortex tissues. Data are presented as mean ± SEM, *n* = 5/group.

**Figure 7.**
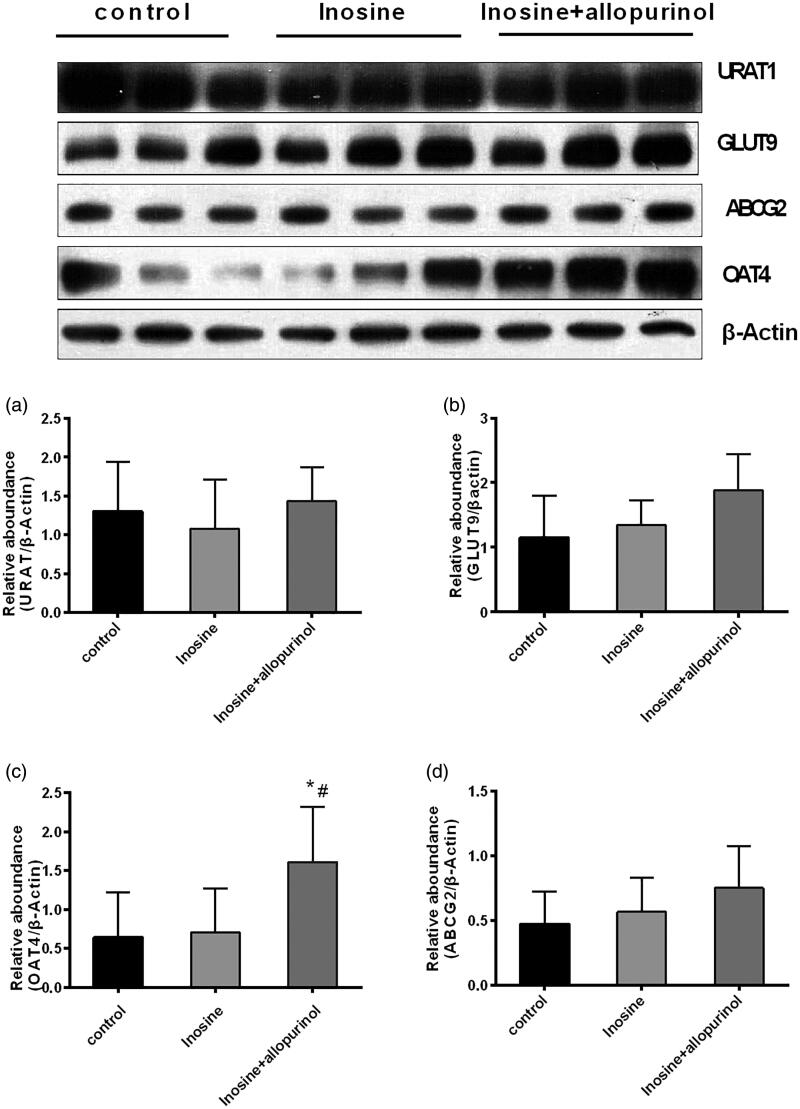
Western blotting analysis of URAT1, GLUT9, OAT4 and ABCG2 in the kidney cortex tissues. Data are presented as mean ± SEM, *n* = 5/group. ^#^*p*< 0.05 vs. 200 mg/kg inosine-treated hyperuricemic monkeys. *p< 0.05 vs. control monkeys.

Inosine administration did not affect the renal transporters URAT1, GLUT9 and ABCG2 at the mRNA and protein levels at 1 h after the injection of 200 mg/kg inosine, as revealed by the qPCR and Western blotting analyses (*p*> 0.05) (Figures 6 and 7).

## Discussion

As a key precursor in purine metabolism, inosine is metabolized by PNP to hypoxanthine, which is subsequently metabolized by XO to xanthine, generating uric acid. Potassium oxonate, a selective competitive inhibitor of uricase, has been used previously to induce HUA in rodents for drug screening (Stavric et al. 1975; Etani et al. 2016). Although rodents are the most commonly used models of HUA, the end product of purine metabolism in these animals is allantoin, which differs from that in humans (and other primates), where (because of the absence of urate oxidase) the final product is urate (Fujiwara et al. 1987; Oda et al. 2002). Hence, primates are more suitable as a model of HUA, but are not widely used (Fanelli and Beyer 1974; Zhu et al. 2017), mainly because primate animal models are costly and the technology required is complex. There are no reports demonstrating the suitability of primates as animal models of HUA.

A retrospective study of clinical cases showed that long-term (average, 4 years) intake of inosine in 30 male patients caused an increase in the SUA level, resulting in asymptomatic HUA. However, the SUA level returned to the normal level after the withdrawal of inosine (Sun 1994). Based on above report, a HUA rodent model was established in mice by sequential injections of potassium oxonate in subcutaneous and inosine in intraparietal respectively. (Yang et al. 2017) To the best of our knowledge, primate animal experiments involving inosine intake have not been reported. Therefore, we investigated inosine-induced HUA in rhesus monkeys.

Epidemiological studies have shown that the level of blood uric acid in a population is related to the sex and age of individuals. The incidence of HUA in men is higher than that in women (Du et al. 1998). This may be closely related to the role of female hormones in promoting the excretion of uric acid from urine (Mikuls et al. 2005). Thus, male animals are often selected as animal models of HUA. In this study, we used male rhesus monkeys aged 9–12 years. To induce HUA, inosine was intraperitoneally injected, for more direct and rapid delivery than oral and intragastric routes. After several preliminary experiments using different doses, inosine doses of 75, 100 and 200 mg/kg were selected for dose-dependent experiments, and 200 mg/kg was found to be the optimal dose. Febuxostat, an XO inhibitor, and allopurinol, a selective inhibitor of xanthine oxidoreductase, are commonly used to treat gout and metabolic syndrome caused by HUA. Ulodesine (Diaz-Torne et al. 2015), an inhibitor of purine nucleotide phosphorylase, is a new drug class that blocks the generation of uric acid precursors more efficiently than inhibitors of XO. The administration of uric acid inhibitors to inosine-induced HUA monkeys confirmed that these drugs lowered the SUA level.

We demonstrated that inosine-treated rhesus monkeys exhibit symptoms of HUA. PNP and XO are key enzymes involved in the purine metabolism pathway. Our results showed that the *PNP* mRNA level was increased by inosine compared with saline. The protein level of PNP was not significantly increased in monkeys after treatment. Furthermore, we observed that the XO mRNA and protein levels in the liver were decreased by inosine compared with saline in monkeys after treatment. It is known that XO is the target of allopurinol and febuxostat in lowering the SUA level. In this study, inosine significantly downregulated the expression of XO mRNA and protein. The mechanism underlying these effects of inosine requires further analysis. A possible underlying mechanism is the initially increased SUA inhibits the expression of XO, the key enzyme involved in UA formation to avoid excessive accumulation of SUA, the final product of purine metabolism. Uric acid production and metabolism are complex processes involving various factors that regulate uric acid production in the liver and reabsorption or excretion from the kidneys and gut.

Organisms can positively regulate their enzyme activities in response to the changes in the internal and external environments despite wide fluctuations in the external environment by changing the rate of biochemical reactions depending on their physiological needs (Rodwell and Kennelly 2003). Claude Bernard proposed the conceptual basis for metabolic regulation (Rodwell and Kennelly 2003), that is, living organisms respond in ways that are both quantitatively and temporally appropriate for their survival against multiple challenges posed by the changes in their external and internal environments. Subsequently, Walter Cannon coined the term ‘homeostasis’ to describe the ability of an animal to maintain a constant intracellular environment irrespective of the changes in their external environment. Organisms respond to the changes in their external and internal environments by balanced, coordinated changes in the rates of specific metabolic reactions. Several human diseases, including cancer, diabetes, cystic fibrosis and Alzheimer’s disease, are characterized by regulatory dysfunctions triggered by pathogenic agents or genetic mutations. Furthermore, in the present study, no changes were observed in the mRNA and protein levels of the renal uric acid transporter. Hence, the present animal model may be suitable for investigating pathogenic mechanisms or developing new therapeutic agents, particularly because the symptoms are stable and reproducible. Importantly, rhesus monkeys and humans have similar uric acid metabolism pathways. Accordingly, these animals may be valuable in drug testing, as they responded to XO inhibitors and other drugs that lower the uric acid level or inhibit purine nucleoside phosphatases.

However, HUA animal models without organ damage may prevent studies of chronic diseases. There are advantages and disadvantages to different animal models. Acute HUA is easily restored to baseline level after the withdrawal of stimulators, whereas persistent HUA has serious negative effects on several organs including the kidney, heart and vessels. When developing rational models, researchers should consider the experimental purposes and conditions. HUA animal models will be valuable for drug testing, as shown by the results showing that they respond to XO inhibitors and other drugs that the lower uric acid level or inhibit purine nucleoside phosphatases.

## Conclusions

Our study suggested that the intraperitoneal injection of inosine would induce a reliable acute HUA symptom in rhesus monkeys, and the model described here appeared to be a rational platform to evaluate therapeutic activities *in vivo* of novel ani-HUA drugs targeting both PNP and XO prior moving to clinic trial, as well as a useful system for HUA pathophysiology studies.
